# Antinociceptive Effect of Intrathecal Injection of Genetically Engineered Human Bone Marrow Stem Cells Expressing the Human Proenkephalin Gene in a Rat Model of Bone Cancer Pain

**DOI:** 10.1155/2017/7346103

**Published:** 2017-02-14

**Authors:** Yi Sun, Yuke Tian, Haifeng Li, Dengwen Zhang, Qiang Sun

**Affiliations:** ^1^Department of Anesthesiology, Guangdong General Hospital and Guangdong Academy of Medical Sciences, Guangdong Sheng, China; ^2^Department of Anesthesiology, Tongji Hospital Affiliated to Tongji Medical College, Huazhong University of Science and Technology, Anhui Sheng, China

## Abstract

*Background*. This study aimed to investigate the use of human bone marrow mesenchymal stem cells (hBMSCs) genetically engineered with the human proenkephalin (hPPE) gene to treat bone cancer pain (BCP) in a rat model.* Methods*. Primary cultured hBMSCs were passaged and modified with hPPE, and the cell suspensions (6 × 10^6^) were then intrathecally injected into a rat model of BCP. Paw mechanical withdrawal threshold (PMWT) was measured before and after BCP. The effects of hPPE gene transfer on hBMSC bioactivity were analyzed in vitro and in vivo.* Results*. No changes were observed in the surface phenotypes and differentiation of hBMSCs after gene transfer. The hPPE-hBMSC group showed improved PMWT values on the ipsilateral side of rats with BCP from day 12 postoperatively, and the analgesic effect was reversed by naloxone. The levels of proinflammatory cytokines such as IL-1*β* and IL-6 were ameliorated, and leucine-enkephalin (L-EK) secretion was augmented, in the hPPE-engineered hBMSC group.* Conclusion*. The intrathecal administration of BMSCs modified with the hPPE gene can effectively relieve pain caused by bone cancer in rats and might be a potentially therapeutic tool for cancer-related pain in humans.

## 1. Introduction

Cancer-related pain is extremely troubling not only for patients but also for their families, often being one of the most burdensome symptoms experienced by cancer patients and severely influencing their quality of life. According to a recent systematic review, approximately 60% of advanced cancer patients suffer from severe cancer-related pain [1]. In another report, 30–50% of patients in the early stage of cancer and 70–90% in the late stage were described as experiencing substantial and intractable pain [2]. However, there is currently no safe and efficacious therapy to eliminate the suffering caused by pain. Three-step ladder treatment is recommended by the World Health Organization (WHO), which provides some instructions on treating cancer-related pain, but drugs typically have an insufficient analgesic effect on severe pain. An investigation showed that approximately 32% of patients receiving such therapy complained of its unsatisfactory therapeutic effects [3, 4]. Poor analgesic efficiency and notable adverse effects continue to severely reduce patient quality of life and are significant problems that need to be resolved. Clearly, the development of more effective treatment for cancer pain is at the top of the “pain relief” list.

Over the last decade, the focus in treating cancer-related pain has shifted beyond drug therapy to novel molecular approaches. These methods, including cell transplantation and gene therapy, can overcome the inefficiency and side effects associated with traditional medicine and provide new therapeutic options for severe pain relief. Bone marrow stem cells (BMSCs) are considered especially promising in the pain-care field. Detailed studies on BMSCs have progressed in many fields and have provided good results. Owing to their remarkable characteristics, increasing attention is being focused on novel uses for these cells. Recently, BMSCs have been shown to exert a tumoricidal bystander effect in suicide gene therapy [5] and selectively deliver therapeutic genes to tumor cells to express an appropriate transgene at tumor loci [6]. Apart from some well-known properties such as being available for harvesting from autologous donors, rapid expansion in vitro, migration to sites of tissue injury, differentiation into neural cells [7], self-renewing capacity, and immunosuppressive features for heterologous transplantation [8], BMSCs have two extraordinary characteristics of particular interest, namely, their ability to maintain genetic stability after gene transfection [9] and to mediate the secretion of a broad range of bioactive molecules [10].

The transplantation of transgenic human BMSCs could be a safe and effective method to relieve cancer-related pain; however, more experiments on animal models are needed, and the safety and efficiency of hBMSC transplantation should be evaluated. In this study, the human proenkephalin (hPPE) gene, which is a classical tool widely used in previous studies [11] on transgenic analgesia, was chosen as a target gene to be transfected into human bone marrow mesenchymal stem cells (hBMSCs), followed by intrathecal injection into a rat bone cancer pain (BCP) model [12]. The antinociceptive effects and biological characteristics of the engineered hBMSCs were then evaluated. This study may be the first to report on the use of modified hBMSCs to treat cancer pain and is instructive for progress in analgesic research.

## 2. Materials and Methods

### 2.1. In Vitro Characterization

#### 2.1.1. hBMSCs Preparation and Differentiation Analysis

hBMSCs were obtained from 4 healthy female donors (age range, 20–45 years) undergoing plastic surgery and after informed consent and authorization from the Hospital Ethical Committee. Primary hBMSCs were cultured and passaged in 75 cm^2^ culture flasks at 37°C in a 5% CO2 humidified incubation chamber. The third passage was utilized for the experiments. The viability of cells was measured. After being washed, trypsinized, and centrifuged, a suspension of cells (1 × 10^5^ cells/100 *μ*L PBS) was stained at room temperature for 30 minutes with phycoerythrin- (PE-) labeled rabbit anti-human CD29 (Serotec, Ltd., United Kingdom), fluorescein isothiocyanate- (FITC-) conjugated anti-human CD44 antibody (Serotec, Ltd., United Kingdom), PE-labeled rabbit anti-human CD34 (Serotec, Ltd., United Kingdom), and FITC-conjugated anti-human CD45 antibody (Serotec, Ltd., United Kingdom). The expression of cell surface antigens was assessed by Fluorescence Activated Cell Sorting (FACS) with flow cytometry (FCT, Becton Dickinson Inc., USA). The evaluation of adipogenesis was detected by Oil Red O staining. The differentiation potential for osteogenesis was assessed by the calcium tubercle sodium alizarinsulfonate staining.

#### 2.1.2. Vector Construction and Transfection

hPPE RNA was isolated from minced, ice-cold adrenal pheochromocytoma tissues. Real-time PCR was performed using specific primers designed in Primer 5.0 software (Biosune Biotechnology LTD, Shanghai, China) based on the GenBank sequence (human PEEK, GenBank #NM006211):

  Forward 5′-ATACGAATTCC**ATG**GCGCGGTTCCTGACA-3′;
  Reverse 5′-GCGCGTCGAC**TTA**AAATCTCATAAATCC-3′

EcoRI/SalRI sites were added to the target gene sequence, corresponding to restriction sites present in the plasmid pBABE puro (a gift from Dr. Xiao, Research Center, GAMS). The recombinant viral vector pBABE-hPPE was identified by restriction enzyme analysis and verified by nucleotide sequencing. The plasmid was transfected into 293T cells using Lipofectamine 2000 (Invitrogen, Paisley, United Kingdom) for screening and viral amplification [13]. hBMSCs were infected with virus-containing media, and puromycin-resistant cells were chosen for use. The biological features of the transfected cells, including cell viability, surface marker expressing, and differentiation potential, were measured. mRNA expression was assessed by RT-PCR.

#### 2.1.3. Fluorescence Immunohistochemistry (IHC)

hPPE-hBMSCs were incubated overnight at 4°C with rabbit anti-human leucine-enkephalin (L-EK) antibody (Bicleaf, Shanghai, China) at a dilution of 1 : 400 and were then washed and incubated with goat anti-rabbit secondary antibodies (1 : 500, Abcam, Cambridge, United Kingdom), conjugated with FITC and Y3. After washing completely, 4′6-diamidino-2-phenylindole (DAPI) was used as a nuclear stain. Fluorescence images were collected using an IX71 SIF-2 fluorescence microscope equipped with an Olympus digital camera.

#### 2.1.4. Enzyme Immunoassay Measurement

After cells in all groups were seeded into culture plates, the supernatants were collected, and the L-EK content was measured using an enzyme immunoassay (ELISA) kit (Xitang Biotech, Co., Ltd., Shanghai, China) following the manufacturer's instructions.

### 2.2. Animals and Experimental Protocol

All animal experimental procedures were approved by the Committee of Animal Use for Research and Education of Guangdong Medical Science Institute.

#### 2.2.1. Catheter Implantation and BCP Model Induction

To receive lumbar intrathecal infusion of hBMSCs, female Sprague-Dawley rats (weighing 220–250 g), purchased from Sun Yat-sen University of Medical Sciences Center for Animal Experiments, Guangzhou, China, were implanted with catheters (item #0007150; DURECT). A laminectomy was conducted at the caudal portion of the L3 spinal level and rostral portion of the L4 spinal levels. The dura was incised using a 25-gauge needle, and an intrathecal catheter was introduced into the subdural space over the spinal cord. The proximal part of the catheter was secured to the lumbar muscle to prevent removal. The proximal part of the catheter was introduced subcutaneously through the thoracic area and exited the skin of the interscapular area. The tip was closed with sterile glue. Rats that displayed fresh blood in the cerebrospinal fluid (CSF) or evidence of gross neurological injury were excluded from the experiment. After a 5-day recovery period, rats received BCP surgery. The BCP model was established as follows: rats were anesthetized with sodium pentobarbital (60 mg/kg, i.p.), and a 1 cm rostro-caudal incision was made over the proximal half of the tibia. A 23-gauge needle was inserted into the intramedullary canal of the tibia, approximately 5 mm below the knee joint to create a cavity for the injection of the cells, and a 10 *μ*L volume of Walker 256 mammary gland carcinoma cells (approximately 2 × 10^5^ cells) or cell culture media were injected into the bone cavity. The cavity was sealed using resin cement. Roentgenography of the ipsilateral tibia was performed preoperatively and on postoperative days 7 and 14. Radiographs were taken using a Latheta LCT-200 X-ray imaging system. The protocol was similar to that described previously [14].

#### 2.2.2. Grouping

Rats were divided into 4 groups of 21 rats each as follows: the normal control group, undergoing sham operation; the BCP group, undergoing insertion of a microspinal catheter into the subarachnoid space at the lumbar region and intrathecal delivery of 10 *μ*L of saline on day 11 after operation; the BCP + pBABE-hBMSC group, undergoing intrathecal delivery of pBABE-hBMSCs (6 × 10^6^ cells/10 *μ*L); and the BCP + hPPE-hBMSC group, undergoing intrathecal delivery of hPPE-hBMSCs (6 × 10^6^ cells/10 *μ*L).

#### 2.2.3. Nociceptive Behavior

Mechanical allodynia was assessed using Von Frey filaments [15]. The monofilaments were used from 1.4 g up to 100 g. Each filament was tested five times. Four additional stimulations were determined, and the 50% paw mechanical withdrawal threshold (PMWT) was calculated using the up-down method. PMWT was measured before operation and at 7, 12, 14, 17, and 21 d after operation. On the last day, PMWT was measured before and 30 min after i.p. naloxone (4 mg/kg) [16].

#### 2.2.4. ELISA

On the 14th day after operation, the rats were killed by an overdose of chloral hydrate (350 mg/kg intraperitoneally). The L_3_-L_4_ spinal cord was removed and stored at −80°C until further processing. Frozen spinal cords were homogenized in normal saline (10 *μ*L/mg tissue). After 4000 rpm centrifugation for 15 min at 4°C, the supernatant was used for ELISA. Cytokines (IL-1*β* and IL-6) content was measured using rat-specific ELISA kits (R&D Systems, MN, USA) according to the manufacturer's instructions. The same process was repeated on day 21 after operation, and the content of L-EK was measured by ELISA as described above.

## 3. Statistical Analysis

Data are expressed as means ± SEM. Statistical analysis was performed using repeated measures one-way analysis of variance followed by the least-significant difference (for equal variance) or Dunnett T3 (for unequal variance) test. *P* < 0.05 was considered statistically significant.

## 4. Results

### 4.1. Characterization of hPPE-hBMSCs In Vitro

**Figure 1:**
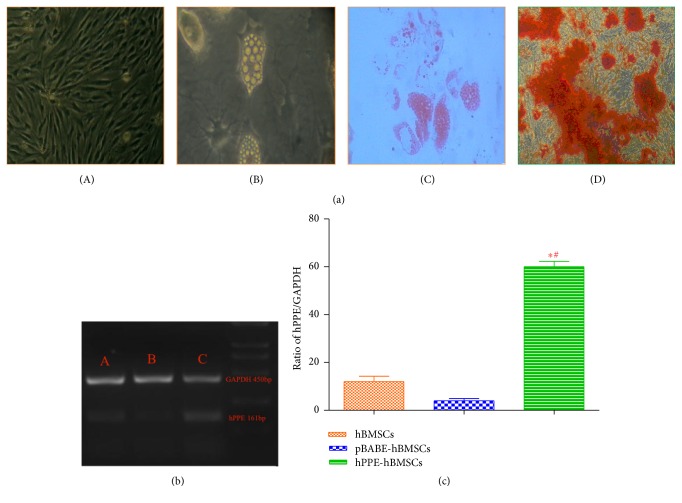
(a) Identification of the human bone marrow stem cells (BMSCs), including culture, differentiation, and application, was analyzed by immunohistochemistry. (A) The morphology of BMSCs at passage 3 (scale bar = 100 *μ*m). (B) Adipogenic differentiation before staining (scale bar = 100 *μ*m). (C) The cultured cells were stained with Oil Red O solution (scale bar = 100 *μ*m). (D) Osteoblasts were stained with alizarin red (scale bar = 100 *μ*m), *n* = 6. (b), (c) The expression of the human proenkephalin (hPPE) gene in engineered hBMSCs (C) was analyzed by RT-PCR 2 weeks (passage 3) after cell transfer. hBMSCs, human bone marrow stem cells; pBABE, a retroviral vector; pBABE-hBMSCs, the pBABE-hBMSC group; hPPE-hBMSCs, the hPPE-hBMSC group. Naïve hBMSCs (A) and vector-engineered hBMSCs (B) served as controls, and hPPE RT-PCR products (161 bp) were expressed as the hPPE/GADPH (450 bp, an internal control) ratio. Statistical analysis showed significantly upregulated hPPE expression in hPPE-hBMSCs compared with hBMSCs and pBABE-hBMSCs (*P* < 0.01). ^*∗*^*P* < 0.01 versus normal hBMSCs and ^#^*P* < 0.01 versus pBABE-hBMSCs, *n* = 6.

Cultured hBMSCs, pBABE-hBMSCs, and hPPE-hBMSCs were CD 29 and 44 positive and CD 34 and 45 negative, with no significant differences between them (*P* > 0.05) (Table 1). hPPE-expressing cells displayed a rapid growth rate up to passage 10, and there were no significant differences regarding the cell activities among the three groups (*P* > 0.05) (Table 2). The proportion of adipocytes after adipoinduction for 3 weeks is shown in Table 3 and Figure 1(a). The results suggested that the cells in all three groups displayed functionally characteristics of multipotential mesenchymal progenitors. There were no significant differences among the three groups (*P* > 0.05), indicating that transfection had no effect on the morphology and proliferation of these cells.

Cultured hBMSCs, pBABE-hBMSCs, and hPPE-hBMSCs at passage 3 were analyzed using FCS for CD 29, 34, 44, and 45. The lack of CD 34 and 45 expressions and the presence of CD 29 and 44 expressions indicate the mesenchymal stem cell lineage of these cells after transfection.

hBMSCs and pBABE-hBMSCs showed low levels of endogenous hPPE gene expression. hPPE-hBMSCs showed a significantly enhanced hPPE gene expression profile compared with the cells in the other two groups (*P* < 0.01) (Figures 1(b) and 1(c)), suggesting that the hPPE gene was integrated into hBMSCs.

**Figure 2:**
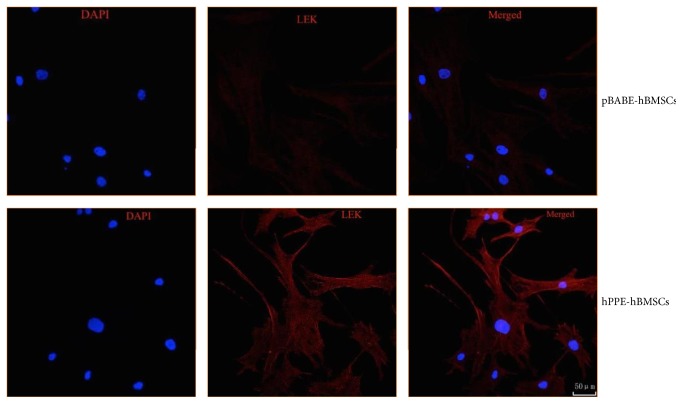
The different expression levels of Leu-enkephalin (L-EK) protein were compared between the pBABE-hBMSC group and the hPPE-hBMSC group by immunofluorescence. pBABE, a retroviral vector; hPPE, human proenkephalin; hBMSCs, human bone marrow stem cells; pBABE-hBMSCs, the pBABE-hBMSC group; hPPE-hBMSCs, the hPPE-hBMSC group. Blue fluorescence marks the nucleus of the hBMSCs by 4′6-diamidino-2-phenylindole (DAPI). Red fluorescence marks L-EK protein. Double-labeled cells, with a blue fluorescent nucleus and a red cytoplasm, represented hBMSCs that expressed the L-EK. There were on differences in nuclear staining between the groups. Little L-EK expression was observed in the pBABE-hBMSC group, whereas greater expression was detected in the hPPE-hBMSC group. All images were obtained with a laser scanning confocal microscope (Leica). Scale bars = 50 *μ*m, *n* = 6.

**Figure 3:**
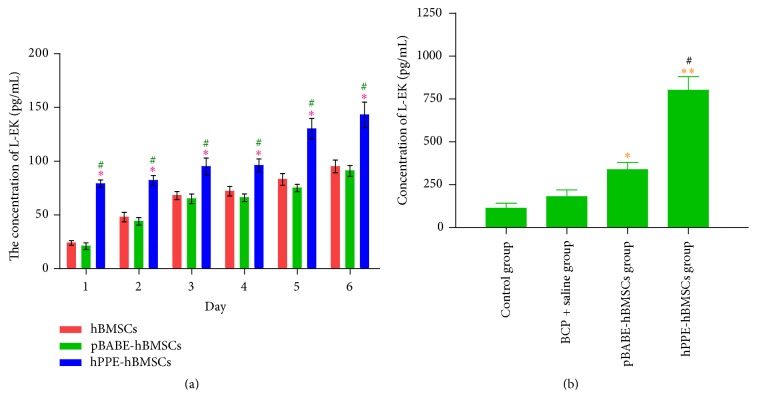
Production of Leu-encephalin (L-EK) by hBMSCs, pBABE-hBMSCs, and hPPE-hBMSCs was measured for 6 days after gene transfection in vitro (a) and in vivo (b). pBABE, a retroviral vector; hPPE, human proenkephalin; pBABE-hBMSCs, the pBABE-hBMSCs group; hPPE-hBMSCs, the hPPE-hBMSCs group; BCP, bone cancer pain; hPPE, human proenkephalin. In (a), from day 1 to day 6, L-EK levels in hPPE gene engineered hBMSCs group were increasing and significantly higher compared with those in the other two groups. The transfection of hPPE accelerated L-EK secretion from hBMSCs. ^*∗*^*P* < 0.05 versus hBMSCs group, ^#^*P* < 0.05 versus pBABE-hBMSCs group. In (b), Leu-encephalin (L-EK) expression was significantly upregulated in human bone marrow stem cells (hBMSCs) transfected with hPPE group and the concentration was higher compared with that in pBABE-hBMSCs (*P* < 0.01) and control (*P* < 0.05) groups. The pBABE-hBMSCs group showed a higher concentration than control group (*P* < 0.05). ^*∗*^*P* < 0.05 versus control group; ^*∗∗*^*P* < 0.01 versus control group; ^#^*P* < 0.01 versus pBABE-hBMSCs group. *n* = 6.

The L-EK protein showed low expressions in the cytoplasm of hBMSCs and pBABE-hBMSCs and was highly expressed in hPPE-expressing hBMSCs (*P* < 0.05), as shown in Figure 2. Naïve hBMSCs, pBABE-hBMSCs, and hPPE-hBMSCs all produced and released L-EK into the culture medium at different levels at each time point, as shown in Figure 3(a). The level of L-EK released by hPPE-hBMSCs was significantly augmented compared with that released by hBMSCs and pBABE-hBMSCs (*P* < 0.05) in serum-free cultures. In addition, L-EK production increased with time, indicating that the reprogrammed cells could survive in the CNS and function normally.

### 4.2. Effect of the Intrathecal Administration of hPPE-hBMSCs

#### 4.2.1. Bone Destruction Evaluation by Radiography

**Figure 4:**
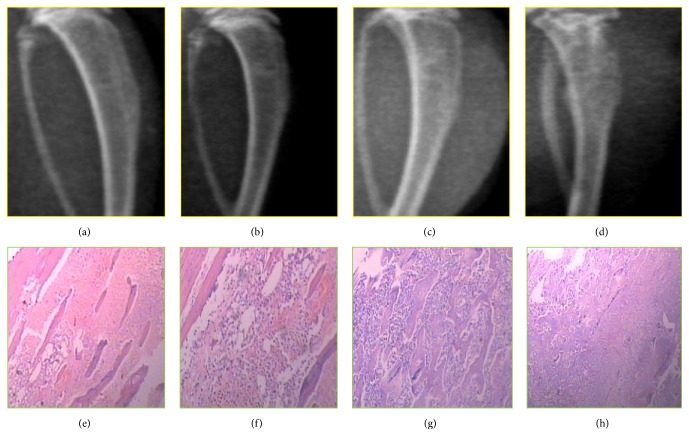
The development of Walker 256 cell-bearing tibias on the ipsilateral side in rats was shown radiologically (a–d) and by immunohistochemistry (e–h). (a) The normal structure of the rat tibia. (b) The evident periosteal reaction was detected 7 d after cancer cell inoculation. (c) Some loss of medullary bone and the erosion of cortical bone were observed 14 d after injection. (d) Significant cortical bone defects were observed 21 d after cancer cell inoculation. Accordingly, hematoxylin and eosin (HE) staining of the tumor area in the rat ipsilateral tibia on different days after operation is shown (e–h). Scale: 50 mm, *n* = 6.

The progressive destruction of the tibia over time after inoculation of Walker 256 cells is shown in Figure 4. No radiological changes were seen in normal bone (Figure 4(a)). A clear periosteal reaction was observed in the proximal epiphysis 7 d after injection (Figure 4(b)). Some loss of medullary bone and erosion of cortical bone arose 14 d after injection (Figure 4(c)). Significant cortical bone defects in the tibia occurred 21 d after injection (Figure 4(d)).

#### 4.2.2. Time Course of Mechanical Allodynia

**Figure 5:**
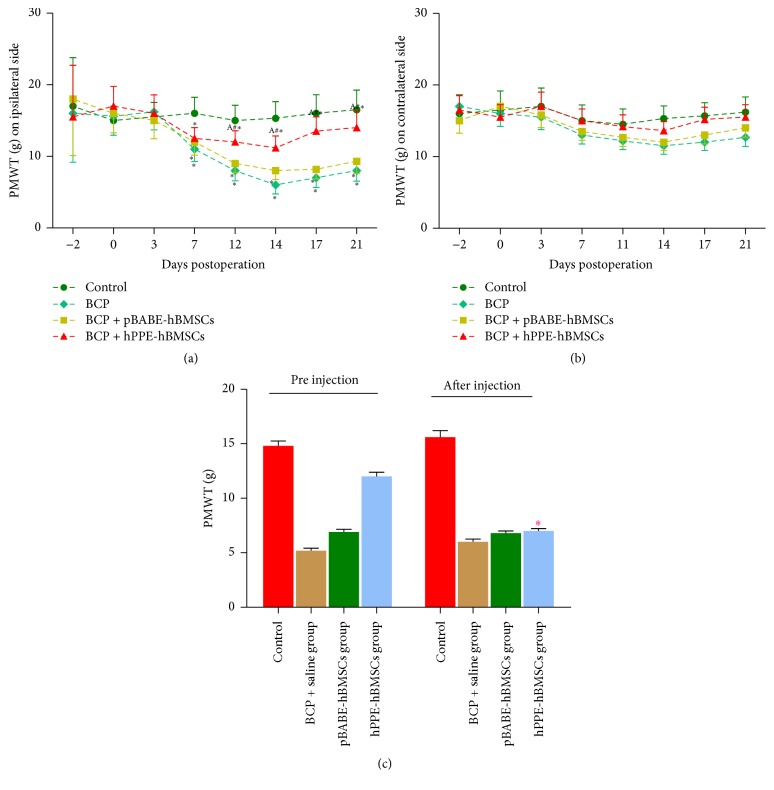
(a) Comparison of the paw mechanical withdrawal threshold (PMWT) indicating mechanical hyperalgesia changes on the ipsilateral side during the time course of Walker 256 cell incursion. Mechanical allodynia occurred starting on day 7, peaked on day 14, and then weakened until day 21. A significant decrease in PMWT was shown in the BCP, BCP + pBABE-hBMSCs, and BCP + hPPE-hBMSCs groups compared with the control group (*P* < 0.05), and treatment with hPPE-hBMSCs impaired the decrease in PMWT caused by cell administration. PMWT in the hPPE-hBMSC group was significantly higher than that in the BCP and pBABE-hBMSC groups (*P* < 0.05). ^*∗*^*P* < 0.05 versus the control group; ^#^*P* < 0.05 versus the BCP group; ^A^*P* < 0.05 versus the pBABE-hBMSC group. BCP, bone cancer pain; hPPE, human proenkephalin; pBABE, a retroviral vector; hBMSCs, human bone marrow stem cells; Control, the control group; BCP, the BCP + saline group; pBABE-hBMSCs, the pBABE-hBMSC group; hPPE-hBMSCs, the hPPE-hBMSCs group. *n* = 6. (b) Comparison of the paw mechanical withdrawal threshold (PMWT) indicating mechanical hyperalgesia changes on the ipsilateral side during the time course of Walker 256 cell incursion. There were no significant differences among the groups (*P* > 0.05). BCP, bone cancer pain; hPPE, human proenkephalin; pBABE, a retroviral vector; hBMSCs, human bone marrow stem cells; Control, the control group; BCP, the BCP + saline group; pBABE-hBMSCs, the pBABE-hBMSC group; hPPE-hBMSCs, the hPPE-hBMSC group, *n* = 6. (c) Naloxone was used to reverse the antiallodynia effect of met-enkephalin in bone cancer rats. The results showed that paw mechanical withdrawal threshold (PMWT) was significantly reduced after naloxone administration (*P* < 0.05). ^*∗*^*P* < 0.05 versus PMWT of the hPPE-hBMSCs group before injection. BCP, bone cancer pain; hPPE, human proenkephalin; pBABE, a retroviral vector; hBMSCs, human bone marrow stem cells. *n* = 6.

In our experiment, mechanical allodynia on both sides was measured before and after the operation. Mechanical allodynia occurred starting on day 7, peaked on day 14, and then decreased until day 21 on the ipsilateral side of the bone cancer. PMWT decreased significantly in the BCP, BCP + pBABE-hBMSC, and BCP + hPPE-hBMSC groups compared with the control group (*P* < 0.05), and hPPE-hBMSC treatment impaired the decrease in PMWT associated with administration. PMWT in the hPPE-hBMSC group was significantly higher than that in the BCP and pBABE-hBMSC groups (*P* < 0.05, Figure 5(a)). hPPE-hBMSC treatment had no significant effect on the contralateral hind paws (*P* > 0.05, Figure 5(b)).

#### 4.2.3. Naloxone Reverses the Analgesic Effect of Met-Enk on Mechanical Hyperalgesia

To determine whether met-enkephalin plays a major role in cancer-related pain relief, naloxone was used to reverse the antiallodynic effect due to met-enkephalin. Thus, naloxone was injected intraperitoneally 30 min after PMWT had been assessed on day 21 after tumor cell inoculation, and PMWT was measured again. PMWT was significantly reduced after naloxone administration (*P* < 0.05). The data also indicated that met-enkephalin secreted by hPPE-hBMSCs might mediate the antiallodynic effect (Figure 5(c)).

#### 4.2.4. IL-1*β* and IL-6 Concentration Measurements

**Figure 6:**
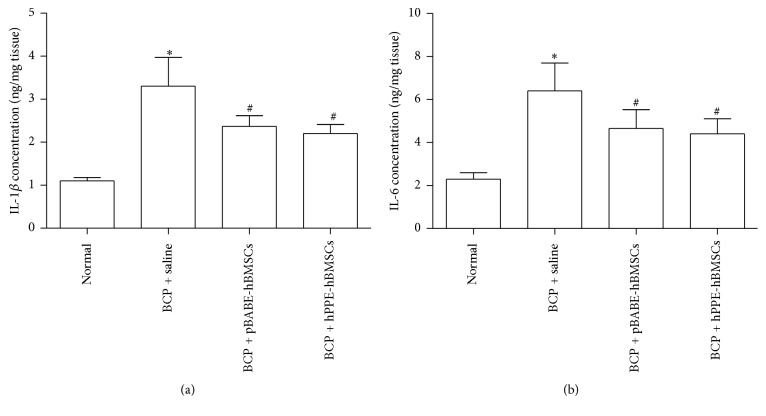
Effects of hPPE-hBMSCs on proinflammatory cytokine levels in the spinal cords of rats with bone cancer pain (BCP) were compared by enzyme-linked immunosorbent assay (ELISA). After treatment with cell transplantation for 3 d following BCP operation, the spinal cords were homogenized and the IL-1*β* (a) and IL-6 (b) levels were measured. Normal, the normal group; BCP + saline, the BCP group; BCP + pBABE-hBMSCs, the BCP + pBABE-hBMSC group; BCP + hPPE-hBMSCs, the BCP + hPPE-hBMSC group. Data were expressed as the mean ± SEM. *n* = 6. Statistical analysis was performed by one-way analysis of variance followed by the least-significant difference or Dunnett T3 test. ^*∗*^*P* < 0.01 versus the normal group; ^#^*P* < 0.05 versus the BCP + saline group. hPPE, human proenkephalin; pBABE, a retroviral vector; hBMSCs, human bone marrow stem cells.

The possible effect of hPPE-hBMSC transplantation on the levels of proinflammatory cytokines in the spinal cord of BCP rats was tested. As shown in Figure 6, no significant differences in the levels of IL-1*β* and IL-6 were found between the BCP and BCP + pBABE-hBMSC groups. Compared with the sham group, IL-1*β* and IL-6 levels were markedly increased in the spinal cords of BCP rats (*P* < 0.001). A significant decrease was observed in IL-1*β* and IL-6 levels after the intrathecal delivery of hBMSCs compared with saline following BCP surgery (*P* < 0.05, Figure 6).

#### 4.2.5. L-EK Level Detection

ELISA revealed that the concentration of L-EK in the spinal cord was higher in the pBABE-hBMSC and hPPE-hBMSC groups than in the control group (*P* < 0.05 and *P* < 0.01, resp.), and the hPPE-hBMSC group demonstrated a significantly higher concentration than the pBABE-hBMSC group (*P* < 0.01), indicating that the L-EK level increased significantly in the spinal cord after hPPE gene transfer (Figure 3(b)).

## 5. Discussion

This study demonstrates the pain-relieving effect of the intrathecal injection of hBMSCs modified with the hPPE gene in a rat bone cancer model. A therapeutic transgene was expressed for an extended time in hBMSCs and induced the enhanced relief provided by L-EK ex vivo and in vivo. The results of a nociceptive behavior test showed that mechanical allodynia occurred on the ipsilateral side in the operation groups compared with the control group. The intrathecal injection of hPPE-hBMSCs significantly increased the withdrawal threshold upon mechanical stimulus on the ipsilateral side compared with the pBABE-hBMSC injection group. The results also showed that hPPE gene transduction not only affected the abilities of hBMSCs to renew themselves or to differentiate but also induced high levels of target gene expression. These findings may have important implications for the use of hBMSCs as vehicles for biological therapeutics, especially for the treatment of cancer-related pain [17].

Cancer-related pain is a severe problem that affects the quality of life of cancer patients, but it cannot be safely and efficiently treated by pharmacological therapies. Even the three-step therapy recommended by the WHO can only partially relieve the pain suffered by cancer patients, but it also has unexpected adverse effects. The promising new approach of gene therapy is now displacing traditional medicine in the treatment of intractable pain, as a result of its reported safety and efficacy. Many previous studies have reported the feasibility and high efficiency of gene therapy in animal models and clinical trials. For example, adult human chromaffin tissue was transplanted and tested in humans for the treatment of terminal cancer pain [18]. Neuronal cells were bioengineered to synthesize and secrete potentially antinociceptive molecules such as the neurotrophin brain-derived neurotrophic factor [19] and the inhibitory neurotransmitter gamma-aminobutyric acid [20], which have been shown to be effective for treating chronic constriction injury (CCI) pain. Although gene therapy appears to have a promising future, some obstacles have not been overcome by current studies, such as the poor efficacy of gene transfer, the extinction of transduced cells, and the intrinsic toxicity of vector systems [21]. A cell-based delivery strategy that exploits the specific properties of BMSCs has the potential to resolve the delivery problems inherent to gene therapy.

As shown in this study, hBMSCs demonstrated particular advantages over the vector cells previously used in pain therapy, which can be summarized as follows: easy collection, rapid expansion, and genetic and phenotypic stability. Our results match those of previous studies [9, 22]. Although hBMSCs have been widely used in many related fields, few applications of them for biological analgesia have been reported. Recently, a single characteristic of hBMSCs was focused on in an attempt to develop a novel method for treating neuropathic pain. Specifically, some studies have revealed the ability of transplanted BMSCs to express the hPPE gene at a low level and to release a homologous analgesic agent to treat neuropathic pain. The therapeutic benefit of this approach was confirmed in an animal model for CCI [23]. Given that (i) cancer-related pain is more severe and intractable than neuropathic pain, (ii) the doses of opiates for cancer pain are ten times higher than those for neuropathic pain [24] in a clinical context, and (iii) transgenic hBMSCs might release a higher level of analgesic agents, hPPE-hBMSCs might be an appropriate subject for study. As expected, our results showed that hPPE-hBMSC therapy had a positive effect, with evident pain reduction. Mechanical allodynia was induced on the seventh day after tumor cell injection. The trend towards decrease in PWMT was inhibited after the intrathecal injection of hPPE-hBMSCs, and the positive effect continued until the end of the experiment, whereas in the pBABE-hBMSC group, the change in PWMT was not significant. HPPE-hBMSCs may behave as “minipumps” to continue producing analgesic agents to suppress the development of severe pain. In previous animal studies, the intravenous delivery of BMSCs had as significant an antinociceptive effect as intrathecal injection. This noninvasive method is clearly promising for research associated with cell transplantation in terms of its safety and convenience, but further consideration of actual results is required. Most hBMSCs were reported to remain in several organs, including the liver, spleen, kidney, and lung, after entering the circulation [25]. Their retention might influence the function of those organs; nevertheless, a few transplanted cells might be able to reach their active site. Extensive research is still required to define the exact mechanisms involved. Conversely, preliminary animal experiments and clinical evidence have suggested that intrathecal injection can provide convincing results [26]. This strategy is promising as a therapeutic method for cell transplantation and was the optimal choice in our study. Otherwise, given its invasiveness, intrathecal injection is more acceptable for cancer pain patients than for those with neuropathic pain.

Cancer-induced pain results from a mixture of mechanisms, including inflammatory, neuropathic, and/or ischemic components [27], of which, the synthesis and release of proinflammatory cytokines such as IL-1*β* and IL-6 may play a pivotal role. Inflammatory processes are involved in both the peripheral and the central nervous system (CNS) and are thought to be involved in the pathogenesis of neuropathic pain [28, 29] because these mediators can further enhance neuroinflammation, thereby leading to the sensitization of nociceptive transmission [30]. In the present study, IL-1*β* and IL-6 levels in the spinal cord of each group were analyzed by ELISA 3 d after hBMSC transplantation, with the hBMSC groups showing more effective therapeutic downregulation of proinflammatory cytokines release. These results are identical to those of previous reports, in which, hBMSCs reduced inflammation by increasing anti-inflammatory cytokines such as interleukin- (IL-) 10 and by decreasing proinflammatory factors such as IL-1*β* and IL-17 [10]. In fact, MSCs are indicated to be the additional important guardian cells for modulating inflammation by recent reports. The role is in part related to their presence as adventitial reticular cells that participate in normal wound repair and in regulation of hematopoietic cells in bone marrow [31]. Recent studies [32] demonstrate the anti-inflammatory effects of MSCs at the cellular and molecular levels from several models of diseases including secreting IL-1 receptor antagonist (IL-1ra) to blunt the effects of IL-1 and tumor necrosis factor-*α* (TNF-*α*); secreting TNF-*α* stimulated gene/protein 6 (TSG-6) to decrease Toll-like receptor 2 (TLR2)/NF-*κ*B signaling in the resident macrophages and increase secretion of prostaglandin E_2_ (PG E_2_) and IL-10. The net effect is decreasing the amplification of the proinflammatory signals and as a result decreasing the recruitment of neutrophils. In this study, the results only confirmed downregulated inflammation by administration of hBMSCs in rat model of bone cancer, but no further exploration was carried out about the possible mechanism. Maybe the verification of anti-inflammation is more important than the discussion on mechanism of inflammation in this study.

hBMSCs relieved mechanical allodynia, and their therapeutic effects against anticancer pain by several methods were highlighted: (i) hBMSCs can secrete neurotrophic factors such as nerve growth factor, basic fibroblast growth factor, and vascular endothelial growth factor; provide protection against nerve damage; and help regenerate and restore damaged nerves [33]. (ii) BMSCs can also modulate spinal cord nociceptive signaling pathways, for example, by blocking the upregulation of inducible nitric oxide synthase expression to suppress pain transmission [34]. (iii) BMSCs have potential inhibitory effects on tumor cell growth in vitro and in vivo by inducing apoptotic cell death and G0/G1 phase arrest in cancer cells [35, 36]. (iv) BMSCs also secrete a certain level of analgesic agents, such as L-EK when including the transgene producing L-EK, to act on the *μ*-opioid receptor, which is the most important of their antinociceptive effects. To determine whether the antinociceptive effects are dominated by the *μ*-receptor activity, the level of mechanical allodynia was estimated after naloxone injection. The significant decrease in PWMT compared with that before injection revealed that the antinociceptive effects were mediated mainly by L-EK released from the transgenic hBMSCs.

In conclusion, this study has demonstrated the possible therapeutic potential of introducing hPPE-expressing hBMSCs to treat cancer-related pain.

## Figures and Tables

**Table 1:** Analysis of cell surface marker expression by flow cytometry after transfection (%, $\bar{X}\;\pm \;SD$).

Group	*N*	CD29	CD44	CD34	CD45
hBMSC P_3_	5	98.10 ± 1.10	94.99 ± 1.00	0.32 ± 0.12	1.78 ± 0.44
hPPE-hBMSC	5	99.08 ± 0.55	95.07 ± 2.35	0.43 ± 0.40	1.48 ± 0.45
pBABE-hBMSC	5	98.99 ± 0.59	93.37 ± 2.36	0.57 ± 0.35	1.82 ± 0.49
*F* value	—	2.368	1.141	0.815	0.796
*P* value	—	0.136	0.352	0.466	0.474

Cultured hBMSC, pBABE-hBMSC, and hPPE-hBMSC at passage 3 were analyzed for CD 29, 34, 44, and 45 expression using FACS. The lack of CD 34 and 45 expression and presence of CD 29 and 44 expressions indicate a mesenchymal stem cell lineage after transfection. hPPE, human proenkephalin; hBMSC, human bone marrow stem cell; pBABE-hBMSC, the pBABE-hBMSC group; hPPE-hBMSCs, the hPPE-hBMSC group.

**Table 2:** Cell activity after freezing and recovering (%, $\bar{X}\pm SD$).

Cell type	hBMSC P_3_	pBABE-hBMSC	hPPE-hBMSC	*F* value	*P* value
Number (*n*)	5	5	5	—	—
Living cell rate (%)	89.90 ± 3.20	87.30 ± 3.26	88.08 ± 2.20	1.033	0.386

There were no significant differences among the three groups (P > 0.05), indicating that transfection had no effect on the morphology and proliferation of these cells. hPPE, human proenkephalin; hBMSC, human bone marrow stem cell; pBABE-hBMSC, the pBABE-hBMSC group; hPPE-hBMSC, the hPPE-hBMSC group.

**Table 3:** Proportion of adipocytes after adipoinduction for 3 weeks (%, $\bar{X}\pm SD$).

Cell type	hBMSC P_3_	pBABE -hBMSC	hPPE-hBMSC	*F* value	*P* value
Number (*n*)	10	10	10	—	—
Ratio of fat cell (%)	77.84 ± 6.40	79.06 ± 7.43	78.88 ± 4.09	0.058	0.944

There were no significant differences in the proportion of adipocytes among the three groups (*P *> 0.05). hBMSC, human bone marrow stem cell; pBABE-hBMSC, the pBABE-hBMSC group; hPPE-hBMSCs, the hPPE-hBMSC group.
